# *N*-(2,6-Dimethyl­phen­yl)maleamic acid

**DOI:** 10.1107/S1600536809042470

**Published:** 2009-10-23

**Authors:** B. Thimme Gowda, Miroslav Tokarčík, Jozef Kožíšek, K. Shakuntala, Hartmut Fuess

**Affiliations:** a Department of Chemistry, Mangalore University, Mangalagangotri 574 199, Mangalore, India; b Faculty of Chemical and Food Technology, Slovak Technical University, Radlinského 9, SK-812 37 Bratislava, Slovak Republic; c Institute of Materials Science, Darmstadt University of Technology, Petersenstrasse 23, D-64287 Darmstadt, Germany

## Abstract

The asymmetric unit of the title compound, C_12_H_13_NO_3_, contains two independent mol­ecules. The conformation of the N—H bond and the C=O bond in the amide segment are *anti* to each other. The mol­ecular conformation of each mol­ecule is stabilized by an intra­molecular O—H⋯O hydrogen bond. In the crystal, mol­ecules are connected through intermolecular N—H⋯O hydrogen bonds. In addition, there is a carbon­yl–carbonyl dipolar inter­action with an O⋯C contact of 2.926 (3) Å.

## Related literature

For our sudies on the effect of ring- and side-chain substitutions on the crystal structures of amides, see: Gowda *et al.* (2009*a* ▶,*b* ▶,*c* ▶); Prasad *et al.* (2002 ▶). For bond-length data, see: Allen *et al.* (1998 ▶). For modes of inter­linking carboxylic acids by hydrogen bonds, see: Leiserowitz (1976 ▶); Jagannathan *et al.* (1994 ▶).
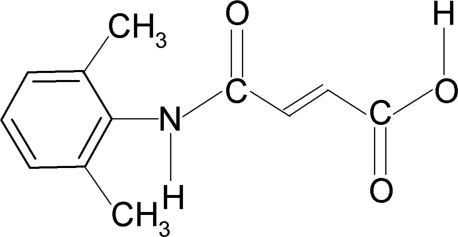


## Experimental

### 

#### Crystal data

C_12_H_13_NO_3_
*M* *_r_* = 219.23
Orthorhombic, 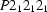

*a* = 12.5268 (4) Å
*b* = 12.9226 (4) Å
*c* = 14.6835 (5) Å
*V* = 2376.95 (13) Å^3^
*Z* = 8
Mo *K*α radiation
μ = 0.09 mm^−1^
*T* = 295 K
0.56 × 0.54 × 0.48 mm

#### Data collection

Oxford Diffraction Xcalibur Ruby Gemini diffractometer
Absorption correction: multi-scan (*CrysAlis Pro*; Oxford Diffraction, 2009 ▶) *T*_min_ = 0.940, *T*_max_ = 0.955
38472 measured reflections
2533 independent reflections
2201 reflections with *I* > 2σ(*I*)
*R*_int_ = 0.025

#### Refinement

*R*[*F* ^2^ > 2σ(*F* ^2^)] = 0.032
*wR*(*F* ^2^) = 0.089
*S* = 1.07
2533 reflections
305 parameters
2 restraints
H atoms treated by a mixture of independent and constrained refinement
Δρ_max_ = 0.14 e Å^−3^
Δρ_min_ = −0.12 e Å^−3^

### 

Data collection: *CrysAlis Pro* (Oxford Diffraction, 2009 ▶); cell refinement: *CrysAlis Pro*; data reduction: *CrysAlis Pro*; program(s) used to solve structure: *SHELXS97* (Sheldrick, 2008 ▶); program(s) used to refine structure: *SHELXL97* (Sheldrick, 2008 ▶); molecular graphics: *ORTEP-3* (Farrugia, 1997 ▶) and *DIAMOND* (Brandenburg, 2002 ▶); software used to prepare material for publication: *SHELXL97*, *PLATON* (Spek, 2009 ▶) and *WinGX* (Farrugia, 1999 ▶).

## Supplementary Material

Crystal structure: contains datablocks global, I. DOI: 10.1107/S1600536809042470/bt5099sup1.cif

Structure factors: contains datablocks I. DOI: 10.1107/S1600536809042470/bt5099Isup2.hkl

Additional supplementary materials:  crystallographic information; 3D view; checkCIF report

## Figures and Tables

**Table 1:** Hydrogen-bond geometry (Å, °)

*D*—H⋯*A*	*D*—H	H⋯*A*	*D*⋯*A*	*D*—H⋯*A*
N1—H1*N*⋯O3^i^	0.87 (2)	2.01 (2)	2.824 (2)	156 (2)
N2—H2*N*⋯O5^ii^	0.839 (19)	2.04 (2)	2.856 (2)	166 (2)
O2—H2*A*⋯O1	0.94 (3)	1.53 (3)	2.465 (2)	173 (2)
O6—H6*A*⋯O4	0.96 (4)	1.52 (4)	2.462 (2)	163 (4)

Symmetry codes: (i) 
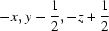
; (ii) 
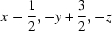
.

**Fig. 1.**
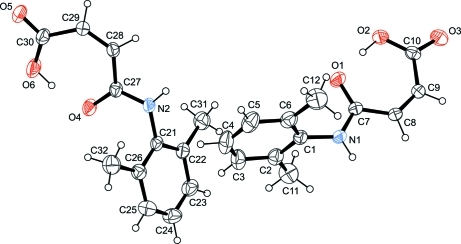
Molecular structure of the two molecules in the asymmetric unit of the title compound showing the atom labelling scheme. Displacement ellipsoids are drawn at the 30% probability level. H atoms are represented as small spheres of arbitrary radii.

**Fig. 2.**
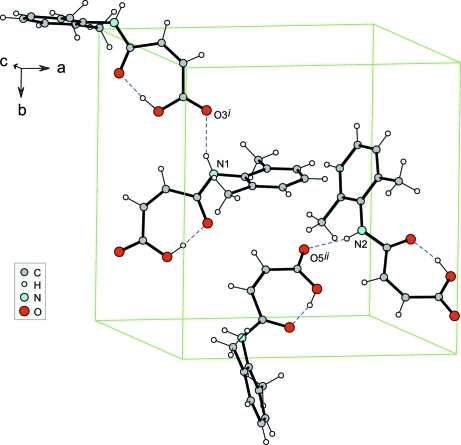
Part of the crystal structure of the title compound with hydrogen bonds shown as dashed lines. Symmetry codes (i): -x,y - 1/2,-z + 1/2; (ii) x - 1/2,-y + 3/2,-z.

## supplementary crystallographic information

### Comment

The amide moiety is an important constituent of many biologically important compounds. As a part of studying the effect of ring and side chain substitutions on the crystal structures of this class of compounds (Gowda *et al.*, 2009*a*,*b*,*c*; Prasad *et al.*, 2002), the crystal structure of *N*-(2,6-dimethylphenyl)-maleamic acid (I) has been determined. The asymmetric unit of the cell contains two independent molecules (Fig. 1). The conformations of the N—H and C=O bonds in the amide segment of the structure are *anti* to each other and those of the amide O atom and the carbonyl O atom of the acid segment are also *anti* to each other. But the amide O atom is *anti* to the H atom attached to the adjacent C atom, while the carboxyl O atom is *syn* to the H atom attached to its adjacent C atom (Fig.1). In the present study, the rare *anti* conformation of the C=O and O—H bonds of the acid group has been observed, similar to that obsrved in *N*-(3,5-dichlorophenyl)succinamic acid (Gowda *et al.*, 2009*c*), but contrary to the more general *syn* conformation observed for C=O and O—H bonds of the acid group in *N*-(2,6-dimethylphenyl)succinamic acid (Gowda *et al.*, 2009*b*). The various modes of interlinking carboxylic acids by hydrogen bonds is described elsewhere (Leiserowitz, 1976). The packing of molecules involving dimeric hydrogen bonded association of each carboxyl group with a centrosymmetrically related neighbor has also been observed (Jagannathan *et al.*, 1994). One intramolecular hydrogen O—H···O bond is present within each maleamic acid moiety. The crystal packing is determined by intermolecular N–H···O hydrogen bonds (Table 1) as seen in Fig. 2. Other intermolecular interactions, which seem to play some role, are the carbonyl-carbonyl interactions, first analyzed in the paper of Allen *et al.* (1998). These dipolar interactions are observed through a short O···C contact of 2.926 (3) Å between the O4 atom of amide moiety in molecule 2 and the C10 atom of the carboxyl moiety in molecule 1 at the position *x* + 1,*y*,*z*. The amido group –NHCO– forms dihedral angles of 80.5 (1)° and 64.0 (2)° with the aromatic ring in the first and second molecules, respectively.

### Experimental

The solution of maleic anhydride (0.025 mol) in toluene (25 ml) was treated dropwise with the solution of 2,6-dimethylaniline (0.025 mol) also in toluene (20 ml) with constant stirring. The resulting mixture was stirred for about 30 min and set aside for an additional 30 min at room temperature for the completion of reaction. The mixture was then treated with dilute hydrochloric acid to remove the unreacted 2,6-dimethylaniline. The resultant solid *N*-(2,6-dimethylphenyl)maleamic acid was filtered under suction and washed thoroughly with water to remove the unreacted maleic anhydride and maleic acid. It was recrystallized to constant melting point from ethanol. The purity of the compound was checked by elemental analysis and characterized by its infrared spectra. The single crystals used in X-ray diffraction studies were grown in an ethanol solution by slow evaporation at room temperature.

### Refinement

C-bonded H atoms were placed in calculated positions with C–H distances of 0.93 Å (C aromatic) and 0.96 Å (C methyl). H atoms attached to nitrogen were refined with the N–H distance restrained to 0.86 (3) Å. The *U*_iso_(H) values were set at 1.2*U*_eq_(C aromatic, N) and 1.5 *U*_eq_(C methyl). The hydroxyl H atoms were freely refined. The absolute structure cannot be determined reliably because anomalous scattering power is too small. In the final refinement cycles the 1938 Friedel pairs  were merged.

### Crystal data

**Table:**

C_12_H_13_NO_3_	*F*(000) = 928
*M**_r_* = 219.23	*D*_x_ = 1.225 Mg m^−^^3^
Orthorhombic, *P*2_1_2_1_2_1_	Mo *K*α radiation, λ = 0.71073 Å
Hall symbol: P 2ac 2ab	Cell parameters from 20238 reflections
*a* = 12.5268 (4) Å	θ = 2.1–29.4°
*b* = 12.9226 (4) Å	µ = 0.09 mm^−^^1^
*c* = 14.6835 (5) Å	*T* = 295 K
*V* = 2376.95 (13) Å^3^	Prism, colourless
*Z* = 8	0.56 × 0.54 × 0.48 mm

### Data collection

**Table:**

Oxford Diffraction Xcalibur Ruby Gemini diffractometer	2533 independent reflections
graphite	2201 reflections with *I* > 2σ(*I*)
Detector resolution: 10.434 pixels mm^-1^	*R*_int_ = 0.025
ω scans	θ_max_ = 25.6°, θ_min_ = 2.1°
Absorption correction: multi-scan (*CrysAlis PRO*; Oxford Diffraction, 2009)	*h* = −15→15
*T*_min_ = 0.940, *T*_max_ = 0.955	*k* = −15→15
38472 measured reflections	*l* = −17→17

### Refinement

**Table:**

Refinement on *F*^2^	Primary atom site location: structure-invariant direct methods
Least-squares matrix: full	Secondary atom site location: difference Fourier map
*R*[*F*^2^ > 2σ(*F*^2^)] = 0.032	Hydrogen site location: inferred from neighbouring sites
*wR*(*F*^2^) = 0.089	H atoms treated by a mixture of independent and constrained refinement
*S* = 1.07	*w* = 1/[σ^2^(*F*_o_^2^) + (0.0646*P*)^2^] where *P* = (*F*_o_^2^ + 2*F*_c_^2^)/3
2533 reflections	(Δ/σ)_max_ < 0.001
305 parameters	Δρ_max_ = 0.14 e Å^−^^3^
2 restraints	Δρ_min_ = −0.12 e Å^−^^3^

### Special details

**Table:**

Geometry. All e.s.d.'s (except the e.s.d. in the dihedral angle between two l.s. planes) are estimated using the full covariance matrix. The cell e.s.d.'s are taken into account individually in the estimation of e.s.d.'s in distances, angles and torsion angles; correlations between e.s.d.'s in cell parameters are only used when they are defined by crystal symmetry. An approximate (isotropic) treatment of cell e.s.d.'s is used for estimating e.s.d.'s involving l.s. planes.
Refinement. Refinement of *F*^2^ against ALL reflections. The weighted *R*-factor *wR* and goodness of fit *S* are based on *F*^2^, conventional *R*-factors *R* are based on *F*, with *F* set to zero for negative *F*^2^. The threshold expression of *F*^2^ > σ(*F*^2^) is used only for calculating *R*-factors(gt) *etc*. and is not relevant to the choice of reflections for refinement. *R*-factors based on *F*^2^ are statistically about twice as large as those based on *F*, and *R*- factors based on ALL data will be even larger.

### Fractional atomic coordinates and isotropic or equivalent isotropic displacement parameters (Å^2^)

**Table:**

	*x*	*y*	*z*	*U*_iso_*/*U*_eq_	
C1	0.25341 (16)	0.39170 (14)	0.10573 (14)	0.0489 (5)	
C2	0.34832 (18)	0.37146 (16)	0.15245 (15)	0.0570 (5)	
C3	0.44161 (19)	0.3689 (2)	0.10282 (19)	0.0703 (7)	
H3	0.5057	0.3534	0.1317	0.084*	
C4	0.4408 (2)	0.3891 (2)	0.0114 (2)	0.0824 (8)	
H4	0.5045	0.3875	−0.0211	0.099*	
C5	0.3477 (2)	0.4115 (2)	−0.03281 (17)	0.0767 (7)	
H5	0.3494	0.4271	−0.0946	0.092*	
C6	0.2509 (2)	0.41152 (16)	0.01250 (16)	0.0617 (6)	
C7	0.10578 (16)	0.47012 (14)	0.18861 (14)	0.0486 (5)	
C8	0.00863 (15)	0.44838 (14)	0.24183 (14)	0.0475 (4)	
H8	−0.0079	0.3787	0.2493	0.057*	
C9	−0.05843 (15)	0.51409 (14)	0.28054 (14)	0.0469 (5)	
H9	−0.1152	0.4829	0.3107	0.056*	
C10	−0.05898 (16)	0.62944 (15)	0.28426 (14)	0.0483 (5)	
C11	0.3486 (3)	0.3555 (2)	0.25411 (18)	0.0814 (7)	
H11A	0.4185	0.3342	0.2734	0.122*	
H11B	0.2976	0.303	0.2698	0.122*	
H11C	0.3299	0.4191	0.2839	0.122*	
C12	0.1475 (3)	0.4315 (3)	−0.0362 (2)	0.0929 (9)	
H12A	0.1169	0.495	−0.0146	0.139*	
H12B	0.0989	0.3755	−0.0246	0.139*	
H12C	0.1605	0.4365	−0.1005	0.139*	
N1	0.15528 (13)	0.38779 (12)	0.15561 (12)	0.0514 (4)	
H1N	0.1307 (18)	0.3268 (17)	0.1688 (16)	0.062*	
O1	0.14060 (13)	0.55904 (11)	0.17420 (12)	0.0726 (5)	
O2	0.01844 (12)	0.68259 (10)	0.24839 (12)	0.0591 (4)	
H2A	0.067 (2)	0.640 (2)	0.2168 (18)	0.071*	
O3	−0.13208 (14)	0.67180 (12)	0.32315 (12)	0.0728 (5)	
C21	0.69266 (14)	0.49032 (15)	0.15611 (12)	0.0432 (4)	
C22	0.64858 (15)	0.48672 (16)	0.24349 (13)	0.0500 (5)	
C23	0.65844 (19)	0.39448 (19)	0.29159 (15)	0.0641 (6)	
H23	0.6292	0.3896	0.3496	0.077*	
C24	0.7103 (2)	0.3108 (2)	0.25536 (18)	0.0734 (7)	
H24	0.7175	0.2504	0.2893	0.088*	
C25	0.7512 (2)	0.31594 (19)	0.16950 (16)	0.0676 (6)	
H25	0.7851	0.2581	0.1453	0.081*	
C26	0.74363 (16)	0.40489 (16)	0.11727 (14)	0.0517 (5)	
C27	0.76542 (15)	0.64136 (16)	0.07866 (12)	0.0452 (4)	
C28	0.73911 (15)	0.73479 (15)	0.02517 (12)	0.0455 (4)	
H28	0.6669	0.7505	0.0205	0.055*	
C29	0.80542 (15)	0.79940 (14)	−0.01721 (13)	0.0459 (4)	
H29	0.7718	0.8548	−0.0457	0.055*	
C30	0.92344 (16)	0.79890 (17)	−0.02710 (16)	0.0556 (5)	
C31	0.5931 (2)	0.5778 (2)	0.28480 (16)	0.0700 (6)	
H31A	0.5664	0.5593	0.3439	0.105*	
H31B	0.5347	0.5982	0.2465	0.105*	
H31C	0.6425	0.6342	0.2906	0.105*	
C32	0.7872 (2)	0.4054 (2)	0.02141 (17)	0.0750 (7)	
H32A	0.8546	0.4408	0.0205	0.112*	
H32B	0.7379	0.4403	−0.0181	0.112*	
H32C	0.7969	0.3355	0.0009	0.112*	
N2	0.68287 (12)	0.58427 (13)	0.10535 (11)	0.0455 (4)	
H2N	0.6232 (16)	0.6091 (17)	0.0914 (15)	0.055*	
O4	0.85813 (11)	0.61612 (13)	0.09830 (11)	0.0665 (4)	
O5	0.96486 (13)	0.85754 (14)	−0.08053 (13)	0.0768 (5)	
O6	0.98128 (12)	0.73556 (19)	0.02073 (15)	0.0970 (8)	
H6A	0.944 (3)	0.685 (3)	0.057 (3)	0.116*	

### Atomic displacement parameters (Å^2^)

**Table:**

	*U*^11^	*U*^22^	*U*^33^	*U*^12^	*U*^13^	*U*^23^
C1	0.0508 (11)	0.0374 (9)	0.0585 (11)	−0.0027 (9)	0.0129 (10)	−0.0054 (9)
C2	0.0592 (12)	0.0494 (12)	0.0623 (12)	−0.0093 (10)	0.0059 (11)	−0.0118 (10)
C3	0.0507 (12)	0.0769 (16)	0.0835 (17)	−0.0117 (12)	0.0087 (12)	−0.0201 (14)
C4	0.0672 (16)	0.0939 (19)	0.0861 (19)	−0.0245 (15)	0.0330 (15)	−0.0243 (16)
C5	0.0903 (19)	0.0815 (17)	0.0581 (13)	−0.0216 (15)	0.0227 (14)	−0.0082 (12)
C6	0.0706 (14)	0.0523 (12)	0.0622 (13)	−0.0056 (11)	0.0079 (12)	−0.0023 (10)
C7	0.0476 (11)	0.0342 (10)	0.0641 (12)	−0.0011 (9)	0.0063 (9)	0.0024 (9)
C8	0.0450 (10)	0.0319 (9)	0.0654 (11)	−0.0024 (8)	0.0027 (9)	0.0004 (9)
C9	0.0408 (10)	0.0411 (10)	0.0587 (11)	−0.0007 (8)	0.0003 (9)	0.0018 (9)
C10	0.0509 (11)	0.0404 (10)	0.0536 (11)	0.0064 (10)	−0.0065 (10)	−0.0059 (9)
C11	0.0836 (17)	0.0905 (18)	0.0702 (15)	0.0041 (15)	−0.0008 (14)	−0.0064 (14)
C12	0.096 (2)	0.107 (2)	0.0757 (17)	0.0050 (18)	−0.0048 (17)	0.0136 (16)
N1	0.0520 (9)	0.0336 (8)	0.0688 (10)	−0.0028 (7)	0.0148 (8)	0.0015 (8)
O1	0.0681 (10)	0.0378 (7)	0.1120 (13)	−0.0057 (7)	0.0323 (10)	0.0003 (8)
O2	0.0579 (9)	0.0351 (7)	0.0843 (10)	−0.0007 (7)	0.0032 (8)	−0.0074 (7)
O3	0.0757 (11)	0.0502 (8)	0.0924 (12)	0.0134 (8)	0.0203 (10)	−0.0100 (8)
C21	0.0318 (8)	0.0537 (11)	0.0440 (9)	−0.0060 (8)	−0.0052 (8)	0.0082 (8)
C22	0.0418 (10)	0.0618 (12)	0.0464 (10)	−0.0055 (9)	−0.0012 (9)	0.0078 (9)
C23	0.0655 (14)	0.0739 (14)	0.0528 (11)	−0.0056 (13)	−0.0001 (11)	0.0197 (11)
C24	0.0832 (17)	0.0629 (14)	0.0742 (15)	0.0040 (13)	−0.0054 (14)	0.0225 (13)
C25	0.0729 (14)	0.0562 (12)	0.0738 (14)	0.0096 (12)	−0.0027 (14)	0.0035 (11)
C26	0.0468 (10)	0.0558 (11)	0.0525 (10)	−0.0011 (10)	−0.0035 (9)	0.0008 (9)
C27	0.0366 (10)	0.0582 (11)	0.0407 (9)	−0.0030 (9)	−0.0040 (8)	0.0062 (8)
C28	0.0344 (9)	0.0538 (11)	0.0484 (10)	0.0008 (9)	0.0007 (9)	0.0040 (9)
C29	0.0424 (9)	0.0460 (10)	0.0494 (10)	−0.0002 (8)	0.0023 (9)	0.0035 (9)
C30	0.0449 (11)	0.0578 (12)	0.0643 (12)	−0.0118 (10)	0.0003 (10)	0.0091 (11)
C31	0.0711 (14)	0.0823 (16)	0.0565 (12)	0.0068 (13)	0.0147 (11)	0.0056 (12)
C32	0.0820 (17)	0.0803 (16)	0.0626 (13)	0.0058 (14)	0.0126 (13)	−0.0049 (13)
N2	0.0311 (7)	0.0567 (10)	0.0486 (8)	0.0000 (7)	0.0006 (7)	0.0115 (8)
O4	0.0365 (7)	0.0845 (11)	0.0784 (10)	−0.0085 (8)	−0.0110 (7)	0.0377 (9)
O5	0.0529 (9)	0.0795 (11)	0.0979 (13)	−0.0113 (8)	0.0134 (8)	0.0336 (10)
O6	0.0379 (8)	0.1251 (17)	0.1281 (16)	−0.0123 (10)	−0.0069 (9)	0.0741 (15)

### Geometric parameters (Å, °)

**Table:**

C1—C6	1.393 (3)	C21—C26	1.397 (3)
C1—C2	1.397 (3)	C21—C22	1.398 (3)
C1—N1	1.432 (3)	C21—N2	1.430 (2)
C2—C3	1.378 (3)	C22—C23	1.391 (3)
C2—C11	1.507 (4)	C22—C31	1.495 (3)
C3—C4	1.368 (4)	C23—C24	1.369 (4)
C3—H3	0.93	C23—H23	0.93
C4—C5	1.365 (4)	C24—C25	1.362 (4)
C4—H4	0.93	C24—H24	0.93
C5—C6	1.383 (4)	C25—C26	1.385 (3)
C5—H5	0.93	C25—H25	0.93
C6—C12	1.502 (4)	C26—C32	1.510 (3)
C7—O1	1.247 (2)	C27—O4	1.240 (2)
C7—N1	1.323 (3)	C27—N2	1.329 (2)
C7—C8	1.473 (3)	C27—C28	1.478 (3)
C8—C9	1.323 (3)	C28—C29	1.332 (3)
C8—H8	0.93	C28—H28	0.93
C9—C10	1.492 (3)	C29—C30	1.486 (3)
C9—H9	0.93	C29—H29	0.93
C10—O3	1.210 (2)	C30—O5	1.208 (2)
C10—O2	1.300 (3)	C30—O6	1.299 (3)
C11—H11A	0.96	C31—H31A	0.96
C11—H11B	0.96	C31—H31B	0.96
C11—H11C	0.96	C31—H31C	0.96
C12—H12A	0.96	C32—H32A	0.96
C12—H12B	0.96	C32—H32B	0.96
C12—H12C	0.96	C32—H32C	0.96
N1—H1N	0.87 (2)	N2—H2N	0.839 (19)
O2—H2A	0.94 (3)	O6—H6A	0.96 (4)
			
C6—C1—C2	122.42 (19)	C26—C21—C22	121.93 (17)
C6—C1—N1	119.32 (19)	C26—C21—N2	119.83 (16)
C2—C1—N1	118.21 (18)	C22—C21—N2	118.22 (18)
C3—C2—C1	117.8 (2)	C23—C22—C21	117.4 (2)
C3—C2—C11	121.3 (2)	C23—C22—C31	120.63 (18)
C1—C2—C11	120.9 (2)	C21—C22—C31	122.01 (18)
C4—C3—C2	120.5 (3)	C24—C23—C22	121.5 (2)
C4—C3—H3	119.7	C24—C23—H23	119.3
C2—C3—H3	119.7	C22—C23—H23	119.3
C5—C4—C3	120.9 (2)	C25—C24—C23	119.9 (2)
C5—C4—H4	119.5	C25—C24—H24	120
C3—C4—H4	119.5	C23—C24—H24	120
C4—C5—C6	121.3 (2)	C24—C25—C26	121.8 (2)
C4—C5—H5	119.3	C24—C25—H25	119.1
C6—C5—H5	119.3	C26—C25—H25	119.1
C5—C6—C1	116.9 (2)	C25—C26—C21	117.45 (19)
C5—C6—C12	121.8 (2)	C25—C26—C32	119.7 (2)
C1—C6—C12	121.3 (2)	C21—C26—C32	122.85 (19)
O1—C7—N1	120.98 (18)	O4—C27—N2	120.93 (17)
O1—C7—C8	123.68 (17)	O4—C27—C28	123.18 (17)
N1—C7—C8	115.34 (16)	N2—C27—C28	115.89 (16)
C9—C8—C7	129.05 (17)	C29—C28—C27	128.42 (18)
C9—C8—H8	115.5	C29—C28—H28	115.8
C7—C8—H8	115.5	C27—C28—H28	115.8
C8—C9—C10	131.31 (19)	C28—C29—C30	131.57 (18)
C8—C9—H9	114.3	C28—C29—H29	114.2
C10—C9—H9	114.3	C30—C29—H29	114.2
O3—C10—O2	121.11 (18)	O5—C30—O6	120.45 (19)
O3—C10—C9	118.2 (2)	O5—C30—C29	119.2 (2)
O2—C10—C9	120.65 (18)	O6—C30—C29	120.32 (19)
C2—C11—H11A	109.5	C22—C31—H31A	109.5
C2—C11—H11B	109.5	C22—C31—H31B	109.5
H11A—C11—H11B	109.5	H31A—C31—H31B	109.5
C2—C11—H11C	109.5	C22—C31—H31C	109.5
H11A—C11—H11C	109.5	H31A—C31—H31C	109.5
H11B—C11—H11C	109.5	H31B—C31—H31C	109.5
C6—C12—H12A	109.5	C26—C32—H32A	109.5
C6—C12—H12B	109.5	C26—C32—H32B	109.5
H12A—C12—H12B	109.5	H32A—C32—H32B	109.5
C6—C12—H12C	109.5	C26—C32—H32C	109.5
H12A—C12—H12C	109.5	H32A—C32—H32C	109.5
H12B—C12—H12C	109.5	H32B—C32—H32C	109.5
C7—N1—C1	124.14 (16)	C27—N2—C21	123.92 (16)
C7—N1—H1N	118.8 (16)	C27—N2—H2N	114.2 (16)
C1—N1—H1N	116.8 (15)	C21—N2—H2N	121.9 (16)
C10—O2—H2A	111.5 (15)	C30—O6—H6A	117 (2)
			
C6—C1—C2—C3	−1.6 (3)	C26—C21—C22—C23	0.8 (3)
N1—C1—C2—C3	175.98 (19)	N2—C21—C22—C23	179.67 (18)
C6—C1—C2—C11	177.0 (2)	C26—C21—C22—C31	−179.38 (19)
N1—C1—C2—C11	−5.4 (3)	N2—C21—C22—C31	−0.5 (3)
C1—C2—C3—C4	2.1 (3)	C21—C22—C23—C24	0.7 (3)
C11—C2—C3—C4	−176.5 (3)	C31—C22—C23—C24	−179.1 (2)
C2—C3—C4—C5	−0.4 (4)	C22—C23—C24—C25	−1.7 (4)
C3—C4—C5—C6	−2.0 (4)	C23—C24—C25—C26	1.1 (4)
C4—C5—C6—C1	2.5 (4)	C24—C25—C26—C21	0.3 (4)
C4—C5—C6—C12	−177.3 (3)	C24—C25—C26—C32	−178.1 (2)
C2—C1—C6—C5	−0.7 (3)	C22—C21—C26—C25	−1.3 (3)
N1—C1—C6—C5	−178.2 (2)	N2—C21—C26—C25	179.84 (18)
C2—C1—C6—C12	179.1 (2)	C22—C21—C26—C32	177.0 (2)
N1—C1—C6—C12	1.5 (3)	N2—C21—C26—C32	−1.8 (3)
O1—C7—C8—C9	1.8 (4)	O4—C27—C28—C29	7.2 (3)
N1—C7—C8—C9	−177.9 (2)	N2—C27—C28—C29	−172.82 (19)
C7—C8—C9—C10	−0.4 (4)	C27—C28—C29—C30	1.7 (4)
C8—C9—C10—O3	178.4 (2)	C28—C29—C30—O5	168.9 (2)
C8—C9—C10—O2	−3.2 (3)	C28—C29—C30—O6	−10.7 (4)
O1—C7—N1—C1	3.2 (3)	O4—C27—N2—C21	−1.2 (3)
C8—C7—N1—C1	−177.03 (18)	C28—C27—N2—C21	178.79 (17)
C6—C1—N1—C7	−84.2 (3)	C26—C21—N2—C27	−64.1 (3)
C2—C1—N1—C7	98.2 (2)	C22—C21—N2—C27	117.1 (2)

### Hydrogen-bond geometry (Å, °)

**Table:**

*D*—H···*A*	*D*—H	H···*A*	*D*···*A*	*D*—H···*A*
N1—H1N···O3^i^	0.87 (2)	2.01 (2)	2.824 (2)	156 (2)
N2—H2N···O5^ii^	0.84 (2)	2.04 (2)	2.856 (2)	166 (2)
O2—H2A···O1	0.94 (3)	1.53 (3)	2.465 (2)	173 (2)
O6—H6A···O4	0.96 (4)	1.52 (4)	2.462 (2)	163 (4)

Symmetry codes: (i) −*x*, *y*−1/2, −*z*+1/2; (ii) *x*−1/2, −*y*+3/2, −*z*.
